# Genetic Diversity and Transmission Characteristics of Beijing Family Strains of *Mycobacterium tuberculosis* in Peru

**DOI:** 10.1371/journal.pone.0049651

**Published:** 2012-11-21

**Authors:** Tomotada Iwamoto, Louis Grandjean, Kentaro Arikawa, Noriko Nakanishi, Luz Caviedes, Jorge Coronel, Patricia Sheen, Takayuki Wada, Carmen A. Taype, Marie-Anne Shaw, David A. J. Moore, Robert H. Gilman

**Affiliations:** 1 Department of Microbiology, Kobe Institute of Health, Kobe, Japan; 2 Wellcome Centre for Clinical Tropical Medicine, St. Mary’s Campus, Imperial College, London, United Kingdom; 3 Clinical Research Department and TB Centre, London School of Hygiene and Tropical Medicine, London, United Kingdom; 4 Laboratorio de Investigación de Enfermedades Infecciosas, Universidad Peruana Cayetano Heredia, Lima, Peru; 5 Department of Microbiology, Osaka City Institute of Public Health and Environmental Sciences, Osaka, Japan; 6 Faculty of Biological Science, University of Leeds, Leeds, United Kingdom; 7 Department of International Health, Johns Hopkins University, Bloomberg School of Public Health, Baltimore, Maryland, United States of America; St. Petersburg Pasteur Institute, Russian Federation

## Abstract

Beijing family strains of *Mycobacterium tuberculosis* have attracted worldwide attention because of their wide geographical distribution and global emergence. Peru, which has a historical relationship with East Asia, is considered to be a hotspot for Beijing family strains in South America. We aimed to unveil the genetic diversity and transmission characteristics of the Beijing strains in Peru. A total of 200 Beijing family strains were identified from 2140 *M. tuberculosis* isolates obtained in Lima, Peru, between December 2008 and January 2010. Of them, 198 strains were classified into sublineages, on the basis of 10 sets of single nucleotide polymorphisms (SNPs). They were also subjected to variable number tandem-repeat (VNTR) typing using an international standard set of 15 loci (15-MIRU-VNTR) plus 9 additional loci optimized for Beijing strains. An additional 70 Beijing family strains, isolated between 1999 and 2006 in Lima, were also analyzed in order to make a longitudinal comparison. The Beijing family was the third largest spoligotyping clade in Peru. Its population structure, by SNP typing, was characterized by a high frequency of Sequence Type 10 (ST10), which belongs to a modern subfamily of Beijing strains (178/198, 89.9%). Twelve strains belonged to the ancient subfamily (ST3 [n = 3], ST25 [n = 1], ST19 [n = 8]). Overall, the polymorphic information content for each of the 24 loci values was low. The 24 loci VNTR showed a high clustering rate (80.3%) and a high recent transmission index (RTI_n−1_ = 0.707). These strongly suggest the active and on-going transmission of Beijing family strains in the survey area. Notably, 1 VNTR genotype was found to account for 43.9% of the strains. Comparisons with data from East Asia suggested the genotype emerged as a uniquely endemic clone in Peru. A longitudinal comparison revealed the genotype was present in Lima by 1999.

## Introduction

Strains of the Beijing family of *Mycobacterium tuberculosis* (*M. tuberculosis*), first described in 1995 [1], have attracted worldwide attention because of their wide geographical distribution and global emergence. These strains have also been shown to have an endemic prevalence in certain regions, including Asia, except for the Indian subcontinent; northern Eurasia; and South Africa [2], [3], [4], [5], [6], [7]. The lineage has been reported to have caused major outbreaks worldwide, some involving drug-resistant variants [2], [8], [9]. These characteristics suggest that strains belonging to this family might have selective advantages (higher virulence or transmissibility) over other *M. tuberculosis* strains [10], [11], [12], [13]. Therefore, a better understanding of the contribution of the Beijing family to the tuberculosis (TB) pandemic is vital to improve global TB control.

The Beijing family is reported to be phylogenetically divisible into 2 main subfamilies: the modern (typical) and ancient (atypical) subfamilies, [14], [15]. The modern subfamily is highly prevalent in China, western Russia, South Africa, and Thailand [3], [15], [16], [17], whereas the ancient subfamily, with a deleted region of difference (RD) RD181[-] (late ancient type), is endemic in Japan and the ancient subfamily with RD181[+] (early ancient type) is endemic in Korea [4], [18], [19], [20], [21], [22], [23], [24]. Although the reasons for the phylogeographical differences remain elusive, the above trends can be used to assess the influence of the Beijing family strains from East Asian countries, where the prevalence is very high [2], [3], on the prevalence of Beijing family strains in other regions. In fact, a large number of Chinese and Japanese immigrants settled in Peru in 19th century. Korea also has a history of migration to Peru, but this migration has occurred more recently. This historical relationship with East Asia encouraged us to characterize the Beijing family strains in Peru.

Unlike in other South American countries, the proportion of Beijing family strains of *M. tuberculosis* in Peru is exceptionally high [25]. In Brazil, Colombia, Paraguay, Venezuela, Argentina, Chile, and Ecuador the prevalence of Beijing family strains was reported to be less than 1% [25], [26], [27], [28], [29]. However, the prevalence in Peru was 5.9% (11/185) in 1999 [25] and 9.3% (30/323) for samples obtained between 2004 and 2006 [30]. Ritacco et al. [25] speculated that the Beijing family strains were first introduced into Peru, and eventually into other South American countries, when Peru received a significant number of Chinese immigrants in the mid-19th century. This same study also showed considerable diversity in the insertion sequence IS*6110* restriction fragment length polymorphism (RFLP) patterns, supporting the concept of earlier introduction(s) of different ancestral strains during the past 150 years. In addition to the importation of Beijing family strains from Asia to Peru, there is also evidence that the Beijing strains were imported into Europe through a South American route, specifically through Peru [31], [32]. Therefore, Peru can be considered as the South American country that has been most strongly affected by the introduction of Beijing family and is also most commonly associated with the spread of these strains to other South American and European countries.

An in-depth analysis of Beijing family strains in Peru may have a significant impact on the understanding of global epidemics involving the *M. tuberculosis* Beijing family strains. The aims of the current study were to unveil the genetic diversity and transmission characteristics of Beijing family strains of *M. tuberculosis* in Lima, Peru, and to elucidate the probable impact of past immigration from East Asian countries.

## Materials and Methods

### Ethics Statement

Prior to the start of the study, ethical approval was obtained from both Universidad Peruana Cayetano Heredia and Imperial College London and institutional approval was obtained from the Peruvian Ministry of Health. Samples for this study were anonymized.

### Study Samples

The Beijing family strains used in this study were identified on the basis of deletion of the spacers 1–34 assessed by the spoligotyping assay [33]. In order to negate the inclusion of “Pseudo-Beijing strains” [34], all of the strains classified as the early ancient type of Beijing family (RD 181[+]) were subjected to the RD 105 analysis [35].

A total of 200 Beijing family strains were identified from 2140 *M. tuberculosis* isolates obtained through a population-level implementation of a new diagnostic test (MODS, Microscopic Observation Drug Susceptibility) [36], [37], which was conducted in Callao and South Lima between December 2008 and January 2010. Strains from all culture-positive patients with respiratory symptoms in the study area were included and none of the samples were duplicated from a single patient. Of the 200 Beijing family strains, 2 were excluded from this study because of insufficient DNA samples. For the remaining 198 Beijing strains, detailed information is provided in Tables 1 and S1.

**Table 1:** Patient demographics for the Beijing family strains in this study.

Characteristics	No. (%) of isolates	
	Between 2008 and 2010	Between 1999 and 2006
Total	198 (100)	70 (100)
Sex		
Male	133 (67)	45 (64)
Female	63 (32)	25 (36)
Unknown	2 (1)	0 (0)
Age group		
<25	85 (43)	19 (27)
25–34	45 (23)	17 (24)
35–44	32 (16)	8 (11)
45–54	10 (5)	3 (4)
55–64	5 (3)	0 (0)
65+	8 (4)	2 (3)
Unknown	13 (7)	21 (30)
Previous TB		
Yes	55 (28)	ND
No	141 (71)	ND
Unknown	2 (1)	70 (100)
HIV status		
Positive	9 (5)	9 (13)
Negative	188 (95)	35 (50)
Unknown	1 (1)	26 (37)
*M. tuberculosis* resistance		
MDR	17 (9)	10 (14)
not MDR	177 (89)	47 (67)
Unknown	4 (2)	13 (19)

An additional 70 Beijing family strains isolated in Lima between 1999 and 2006 were also analyzed in order to make a longitudinal comparison. In detail, 26 were obtained from a previous study in North Lima between 2004 and 2006 [30] and 44 were obtained from 4 distinct areas in Lima (North, South, East, and Central Lima) through the hospital based studies between 1999 and 2004. The details of the strains are described in Tables 1 and S2.

### VNTR Typing

Genotypic data for the 24 loci that comprised the international standard set of 15 loci of variable number of tandem repeat(s) of mycobacterial interspersed repetitive units (15-MIRU-VNTR) [38], and 9 additional loci (2074, 2372, 3155, 3336, 3232, 3820, 4120, QUB11a, QUB18) were analyzed. The 9 additional loci were selected as Beijing-type optimized loci because of their highly discriminatory values in different studies focused on Beijing family strains [4], [24], [39], [40], [41], [42], [43]. This 24-loci VNTR was called the 24_Beijing_-VNTR. The polymerase chain reaction (PCR) primers and the number of repeats for each locus, based on the *M. tuberculosis* H37Rv strain, are described in Table S3; the PCR conditions were as described previously [4]. The amplicon samples were diluted 20-fold with ultrapure water and analyzed on an AB3500 genetic analyzer system (Applied Biosystems, Foster City, CA) at a constant room temperature of 25°C following the manufacturers’ instruction, i.e., capillary temperature, 60°C; electrophoresis voltage, 8.5 kV; and separation time, 5800 s. A GeneScan 1200 LIZ Size Standard (Applied Biosystems) was used to provide internal size markers. Fragment sizes were measured using GeneMapper Ver. 4 (Applied Biosystems). The numbers of repeats at each locus were calculated using the offset values of the size, which correct differences in relative migration between the size standard and the amplicons depending on the locus. The reproducibility and accuracy of size calling and the size offsets were checked by including *M. tuberculosis* H37Rv and 1 Beijing family strain (reference for quality control) into every batch of the analysis (one 96-well plate was used as a batch). For the large alleles (specifically, for the one larger than 1000 bases) of a locus, we used stutter peak counting, as shown in Figure S1, to obtain unambiguous results with high reproducibility [4]. Moreover, we confirmed the reproducibility of our assay by blindly re-testing 22 selected samples (Table S1). The allelic diversity of each VNTR locus was evaluated using Nei’s diversity index [44], i.e., the polymorphic information content (PIC) (Table S4). Genotypic discrimination of the 198 Beijing strains, based on 15-MIRU-VNTR and 24_Beijing_-VNTR, were calculated using the Hunter-Gaston discriminatory index (HGDI) [45]. A recent transmission index (RTI_n−1_) [46], [47] was also calculated using the VNTR profiles. A minimum spanning tree (MST), based on VNTR types, was constructed using Bionumerics software (Bionumerics ver. 4.2; Applied Math., Sint-Martens-Latem, Belgium), as previously described [23].

### Single Nucleotide Polymorphisms at 10 Loci

The sequence types (ST) were determined based on the 10 synonymous SNPs, which were sufficient to divide the Beijing strains obtained from the global population [48]. Each chromosomal position in the whole-genome sequence of H37Rv [49] was as follows: 797736, 909166, 1477596, 1548149, 1692069, 1892017, 2376135, 2532616, 2825581, and 4137829. The SNP at position 1477596 is the same as *ogt*12, which discriminates between the ancient and modern type Beijing strains [16]. The SNP at 2532616 is the same as *adhE2* (codon 124), which further discriminates the modern type [50]. SNPs at 797736 and 2825581 can discriminate between the early ancient (RD181[+]) and late ancient (RD181[-]) Beijing strains [19]. Polymorphic nucleotides of the respective isolates were determined according to Hanekom et al. [17]. STs were designated according to Filliol et al. [48] and Iwamoto et al. [19].

### Data Retrieved from Previous Reports

15-MIRU-VNTR data and the results of subfamily classifications were retrieved from previous publications for the isolates in China [50], Japan [18], and Korea [20]. In order to compare the population structure, based on Filliol’s STs, further sublineage classifications for the Chinese and Korean isolates were determined according to the phylogenetically informative VNTR loci data proposed by Wada and Iwamoto [51]. The VNTR data from Peru and these 3 East Asian countries were compared by constructing MST, based on 15-VNTR-MIRU, as previously described [23].

### Statistical Analysis

Fisher’s exact test (SPSS 17.0; IBM, NY, USA) was used to determine the association of large cluster-forming isolates with patient gender, history of TB treatment, HIV status, and strain drug susceptibility; the association with the median age of patients was evaluated by Mann-Whitney *U*-test using PASW statistics 18 (IBM).

## Results

### Proportion of Beijing Family Strains in Peru

The Beijing family strain, with a prevalence of 9.3% (200/2140), was the third largest spoligotyping clade after the H3 and T1 clades in the study population sampled between 2008 and 2010. This ratio is the same as for the previous study (9.3%, 30/323) conducted in 2004 and 2006 [30] but higher than in 1999 (5.9%, 11/185) [25]. In another study setting, which collected Beijing strains between 1999 and 2004 from 4 distinct areas in Lima, the ratio was 5% (46/912, 2 Beijing strains were excluded in this study because of the lack of DNA). This would imply an increasing trend of Beijing family strains in Lima, Peru. We negated the existence of “Pseudo-Beijing strains” [34] in our sample set by the detection of RD 105 [-] [35] for all of the strains classified as early ancient type by SNP typing.

### Classification of Beijing Family Strains by SNP Typing and MIRU-VNTR

Further subdivision of the Beijing family strains in Peru, according to the 10 loci SNP panel [17], [19], [48], revealed similar population structures between the 2 sample sets (2008–2010 and 1999–2006) (Table 2). The high prevalence of isolates associated with the modern subfamily in Peru is consistent with the worldwide trend, except in the case of Japan and Korea where the ancient subfamily predominates [20], [50], [51], [52], [53], [54]. When we compared the population structure of the Beijing family strains in Peru with China, Japan, and Korea (Table 2), the characteristics of the Peruvian population structure highlighted a high frequency of the ST10 sublineage. ST19/ST25, which evolved before the modern subfamily [16], [51], predominated in the ancient subfamily, as has been seen in China. Recently, Wada et al. proposed the use of SNP at 1576481 instead of 909166 due to the probable homoplastic behavior of SNP 909166 [55]. This is especially critical for discriminating between STK and ST3, which are highly prevalent in Japan. We, therefore, applied it for 4 ST3 stains in our sample set and confirmed them as true ST3 sublineage. Interestingly, the phylogenetic informativity of certain VNTR loci reported in Asian strains [16], [51] was also retained for the strains in Peru (Table 3). Specifically, VNTR 4156, 1955, and 3155 demonstrated high sensitivity and specificity for the sublineages classification.

**Table 2:** Distribution of Beijing sublineage strains.

SNP type	No. (%) of isolates					Definition^2^
	Peru		Japan	China^1^	Korea^1^	
	2008–2010 (n = 198)	1999–2006 (n = 70)	ref [18] (n = 714)	ref [50] (n = 187)	ref [20] (n = 62)	
ST11	0 (0)	0 (0)	3 (0.4)	9 (4.8)	29 (46.8)	Early ancient
ST26	0 (0)	4 (5.7)	50 (7.0)			
STK	0 (0)	0 (0)	111 (15.5)	0	2 (3.2)	Late ancient
ST3	3 (1.5)	1 (1.4)	182 (25.5)	3 (1.6)	3 (4.8)	
ST25	1 (0.5)	0 (0)	6 (0.8)	52 (27.8)	10 (16.1)	
ST19	8 (4.0)	5 (7.1)	195 (27.3)			
ST10	178 (89.9)	53 (75.7)	135 (18.9)	93 (49.7)	18 (29.0)	Modern
ST22	8 (4.0)	7 (10.0)	32 (4.5)	30 (16.0)		

1 Two isolates in Korea and 4 isolates in China could not be assigned sublineages and excluded from the analysis.

2 Early ancient, RD181 [+]; Late ancient, RD181 [−].

**Table 3:** Specific VNTR allele for Beijing sublineages *M. tuberculosis* in Peru (n = 268).

VNTR locus	Specific allele	Corresponding sublineage(s)	No. of isolates	Sensitivity (%)	Specificity (%)
4156	3	Modern	246	246/246 (100)	246/248 (99.2)
	4	Early Ancient	4	4/4 (100)	4/4 (100)
	5	Late Ancient	16	16/16 (100)	16/18 (88.9)
1955	4	Modern	246	235/246 (95.5)	235/235 (100)
3155	2	Early Ancient	4	4/4 (100)	4/4 (100)

**Figure 1:**
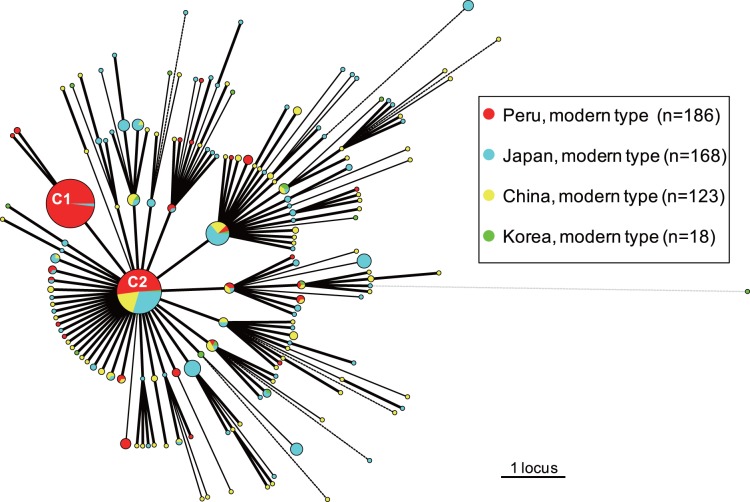
A minimum spanning tree based on 15 loci of a variable number of tandem repeat(s) of mycobacterial interspersed repetitive units (15-MIRU-VNTR) genotyping of the modern subfamily of *M. tuberculosis* Beijing strains from Peru (n = 186), Japan (n = 168), China (n = 123), and Korea (n = 18). Circles correspond to the different types discriminated by 15-MIRU-VNTR genotypes. Their sizes are proportional to the numbers of isolates sharing an identical pattern. The origin of each isolate is represented by different colors. Heavy lines connecting 2 types denote single-locus variants; thin lines connect double-locus variants; and dotted lines (black), triple-locus variants. The gray dotted lines indicate the most likely connection between 2 types differing by more than 3 VNTR loci.

The 15-MIRU-VNTR data from the modern subfamily of Beijing strains in China, Korea, and Japan were retrieved from previous reports [18], [20], [50] and compared with the data of the population-based study in Peru (2008–2010) (Fig. 1). Two extremely large clusters were found in the MST, one (n = 103) composed of almost exclusively Peruvian isolates (101 Peruvian isolates and 2 Japanese isolates) (C1 in Fig. 1) and the other (n = 88) composed of isolates from all of the 4 countries (C2 in Fig. 1). None of the large clusters were composed exclusively of Japanese, Chinese, or Korean isolates. The biggest cluster, C1, did not show a star-like network with the others; rather, it had a nearly terminal topological position. In addition, the VNTR profile could not be found in the MIRU-VNTR*plus* database [56] or in other reports from Russia and China [57], [58]. Together, these observations suggested that this genotype emerged as a uniquely endemic clone in Peru. The other large cluster (C2) was found at the core position and was connected with many isolates, regardless of their geographical origin. Because the Peruvian isolates belonging to the C2 cluster were further subdivided into many different genotypes by the 24_Beijing_-VNTR analysis (data not shown), it is clear that the large cluster was formed because of the convergence of VNTR profiles. Other than these 2 large clusters, all of the other isolates dispersed equally (Fig. 1), i.e., no other branches consisted solely of Peruvian isolates.

### Allelic Diversity of 24 VNTR Loci in Peru

The allelic diversity of each of the 24 VNTR loci is listed in Table S4, including the data from a previous report for Russia, Japan, and China [43]. Overall, the allelic diversity of the VNTR loci in Peru was much lower than in China or Japan. This low diversity was similar to that observed in Russia, where the Beijing family strains are considered to have recently emerged [57], [59]. Only 11 out of the 24 loci had a PIC value greater than 0.1. Of these loci, 7 were in the 9 additional VNTR loci described in the Methods section. The PIC for the 3 hypervariable loci [4] were high: VNTR 3232 (0.710), VNTR 3820 (0.592), and VNTR 4120 (0.495). These results clearly supported the necessity of the use of the 9 additional loci for improving the discriminating power of VNTR genotyping for these strains. As expected, the use of 24_Beijing_-VNTR improved discrimination compared to that of 15-MIRU-VNTR (Table 4).

**Table 4:** Clustering analysis of 198 Beijing family strains (2008–2010) in Peru.

	No. of patterns	No. of clusters	No. clustered isolates	Clustering rate (%)	RTI_n−1_	HGDI
15 VNTR	31	14	181	91.4	0.843	0.688
24 VNTR	58	19	159	80.3	0.707	0.797

### Transmission Characteristics of Beijing Family Strains

**Figure 2:**
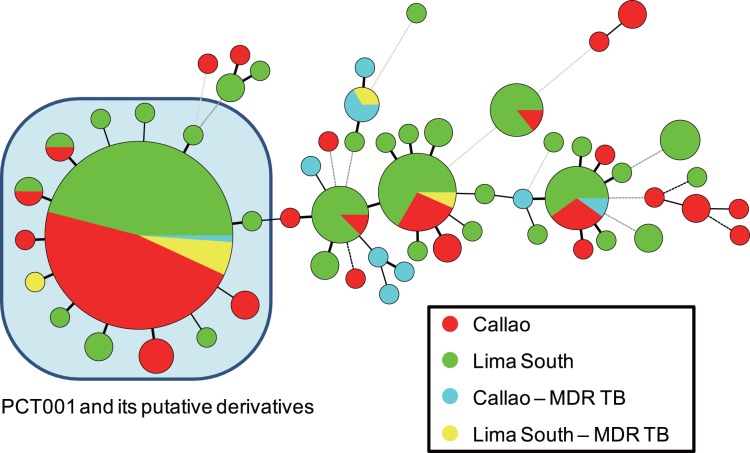
A minimum spanning tree of 198 Beijing family strains from Peru based on the 24-loci variable number of tandem repeats (VNTR). The colors of the circles represent the areas where the strain was isolated and its multidrug-resistant status. The designations for each circle and line in the tree are the same as in Fig. 1.

Although 24_Beijing_-VNTR improved the power of strain discrimination, the results showed high levels of clustering (80.3%) and RTI_n−1_ (0.707) (Table 4). This strongly suggests the active and on-going transmission of Beijing family strains in the survey area. Notably, a large size clustering (43.9%, 87/198) was identified, which was named Peru Cluster Type 001 (PCT001) (Fig. 2). The PCT001 strains belong to the largest cluster (C1) in 15 MIRU-VNTR (Fig. 1), therefore, they are considered a singular genotype in Peru. In the MST (Fig. 2), it formed a star-like VNTR-based network with the probable derivatives from it, mainly resulting from single locus changes. The results suggest the continuous evolution of the clone through active transmission and thus being a “currently successful clone” of the Beijing family strains in Peru. The second and third largest clusters were much smaller than PCT001∶15 isolates (7.6%) and 10 isolates (5.1%), respectively. Although the number of multidrug-resistant *M. tuberculosis* (MDR-TB) belonging to the PCT001 cluster is small at the moment (n = 6), this should be an addressed as a potential public health threat. The comparison between patients harboring the PCT001 strains and those of other patients harboring Beijing strains revealed that only gender is significantly different (*P* = 0.02) (Table 5).

**Table 5:** Demographics of successful clone “PCT001” and other strains.

Variable	No. of strains (%)		*P* value
	PCT001 (n = 87)	Others(n = 111)	
Gender			
Male	67 (77)	66 (59)	0.02
Female	20 (23)	43 (39)	
Unknown	0 (0)	2 (2)	
Median age (range)	23.5 (12–69)	27 (12–83)	0.07
Unknown	4 (5)	9 (8)	
Previous TB			1
Yes	24 (28)	31 (28)	
No	63 (72)	78 (70)	
Unknown	0 (0)	2 (2)	
HIV status			0.30
Positive	2 (2)	7 (6)	
Negative	85 (98)	103 (93)	
Unknown	0 (0)	1 (1)	
MDR TB			0.45
Yes	6 (7)	11 (10)	
No	81 (93)	96 (86)	
Unknown	0 (0)	4 (4)	

**Figure 3:**
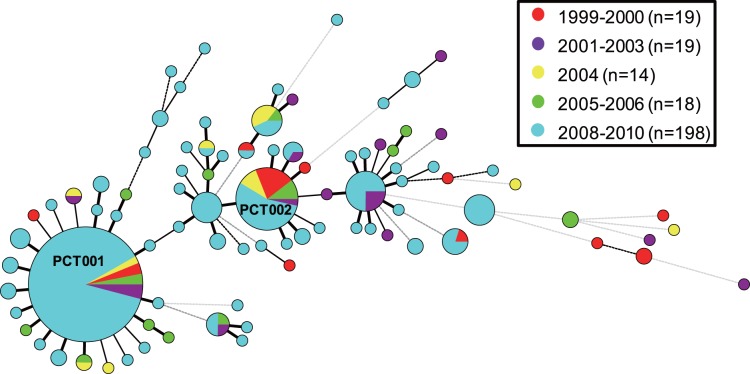
A minimum spanning tree of 268 Beijing family strains comprised of the 198 strains from the population-based study between 2008 and 2010 and an additional 70 strains isolated between 1999 and 2006. The colors of the circles represent the years of isolation. The designations for the circles and lines in the tree are the same as in Fig. 1.

To ascertain whether the PCT001 genotype strains were present before the surveillance periods (in 2008–2010), an MST was constructed based on the 24_Beijing_-VNTR profiles of the 268 Beijing family strains, comprised of the 198 strains (2008–2010) and the additional 70 strains isolated between 1999 and 2006 (Fig. 3). Twelve strains, which formed the second largest cluster among the 70 Beijing family strains, belonged to PCT001 (1 in 1999, 2 in 2000, 4 in 2001, 2 in 2004, and 3 in 2005), indicating that the genotype had existed since at least 1999. A striking difference between the 2 sample sets was found in the ratio of PCT001 genotype strains to total Beijing strains, i.e., a larger cluster (13 strains) than PCT001 was found in the 70 Beijing strains (PCT002 in Fig. 3). When we just focused on the strains isolated in South Lima (24/70 [34.3%]), the overlapping area of these 2 sample sets, PCT002 was still identified as the largest cluster (7 strains) followed by PCT001 (6 strains). These results suggested that the prevalence of PCT001 in the survey area increased over a short period.

## Discussion

The high prevalence of Beijing family strains in Peru (5.9%, 11/185) compared to other South American countries was first reported as the result of a survey of 7 countries on the continent [25]. Very recently, Taype et al. [30] reported that the proportion of TB patients with the Beijing family strains in Peru was 9.3% (30/323). The report nicely described the genetic diversity of all the *M. tuberculosis* in Peru. However, the population size was too small to investigate the Beijing family strains in detail. In the current study, with a much larger population size and a well-designed sample collection scheme between 2008 and 2010, the genetic diversity and transmission dynamics of Beijing strains in Peru was clarified. The results highlighted the following: 1) an increasing prevalence of Beijing family strains during a past decade in Peru, 2) high clonality of the Beijing family strains, suggesting active and ongoing transmission, 3) the successful clone “PCT001 genotype,” which has existed since at least 1999 as a singular clone in Peru, and 4) China as the greatest contributor of imported Beijing family strains into Peru. This study is limited by being unable to determine whether the highly active, ongoing transmission of Beijing family strains is specific for the family or a common trend for all strains in Peru. However, we could find a similarly high clustering rate of Beijing family strains (24/30 [80%]), compared with that of the non-Beijing family strains (174/293 [59.4%]) in the previous study using 12-MIRU-VNTR and spoligotyping [30]. This higher clustering in Beijing family strains implies a high transmission of it in Peru (more successful than non-Beijing strains). This trend could be confirmed later on by further VNTR analysis of all of the 2140 isolates.

A longitudinal comparison with the 70-strain sample set collected between 1999 and 2006 suggested that the successful clone, PCT001, was already present in Lima, by 1999, but at a lower prevalence (n = 12 [17.1%]) (Fig. 3). This difference in the ratio of PCT001 genotype strains to the total Beijing strains between the 2 sample sets suggests that the increase in PCT001 prevalence occurred recently, over a relatively short period. HIV infection, one of the high-risk characteristics for a large-scale outbreak [2], did not explain this high prevalence of PCT001 strains in the survey area (Table 5). One of the possible explanations for the high prevalence of PCT001 strains could be that it is highly transmissible and/or has increased virulence. The highly prevalent strains from large clusters have been previously reported to be more virulent than the sporadic strains of lower prevalence [59], [60]. An attractive hypothesis is that PCT001 strains gained a selective advantage that allowed them to have spread more easily between the 2 sample collection periods (1999–2006 and 2008–2010).

The 15-MIRU-VNTR data from the modern subfamily of Beijing strains in China, Japan, and Korea were retrieved from previous reports [18], [20], [50] for comparison with the Peruvian isolates. The topology of the MST, based on these data (Fig. 1), suggested that the PCT001 genotype was a uniquely endemic clone in Peru. Except for the 2 largest clusters, the VNTR patterns from these countries were similarly diverse and none of the branches was composed exclusively of Peruvian isolates. This result would further support the earlier introduction of different ancestral strains into Peru from Asia during the past 150 years, an idea that was proposed on the basis of the diversity in IS*6110* RFLP patterns in a previous study [25].

The 10 loci SNPs could identify the modern Beijing lineage ST10 as the predominant sublineage in Peru. Very recently, this sublineage was reported as the most common in Taiwan and Thailand [16], [61]. Allelic distribution of VNTR loci in each sequence type of Peruvian samples revealed the phylogenetic informativity of 3 MIRU-VNTR loci (4156, 1955, 3155) (Table 3). This is consistent with the results for East Asian strains [51] and could be an evidence for sharing the common ancestors of the Beijing family strains in Peru with those in East Asian countries. With the tremendous increase in whole-genome sequencing data, an increasing number of SNP typing systems have been developed [54], [62], [63]. The 10 loci SNPs typing is useful for a classification of Beijing family strains into sublineages level but apparently needed more discriminatory power to compensate for the VNTR homoplasy effect (VNTR-based clusters with mixed SNP sublineages), which was demonstrated with optical sets of 8 SNPs in Shanghai [50]. Moreover, inclusion of the lineage specific SNPs for the clades other than Beijing family [62], [63] would expand the potential for phylogenetic studies. Further elaboration and optimization of the SNP sets would facilitate future molecular epidemiology and phylogenetic studies on *M. tuberculosis* in Peru.

In conclusion, the current results revealed the predominance of the modern subfamily and active transmission of Beijing family strains within Peru. Moreover, the emergence of the highly prevalent strains with the PCT001 genotype was also detected. The importation of Beijing family strains into European countries from Peru has already been reported [31], [32], raising concern over transnational transmission. Future trends regarding the prevalence of PCT001 strains and the changes in population structure need to be carefully monitored from both the local and global epidemiological standpoints.

## Supporting Information

Figure S1 **Quality control approach for VNTR.**
(PDF)

Table S1 **Description of** ***M. tuberculosis*** **Beijing family strains obtained between December 2008 and January 2010 in Lima.**
(XLS)

Table S2 **Description of the additional 70** ***M. tuberculosis*** **Beijing family strains in Lima, Peru.**
(XLS)

Table S3 **Locus designations and PCR primer sequences of the VNTR locus.**
(XLS)

Table S4 **Allelic diversity of VNTR loci.**
(XLS)
