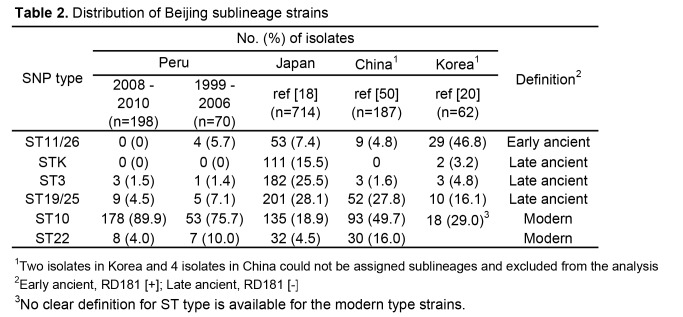# Correction: Genetic Diversity and Transmission Characteristics of Beijing Family Strains of *Mycobacterium tuberculosis* in Peru

**DOI:** 10.1371/annotation/a38090da-2437-4af0-b84a-0bb5de59805f

**Published:** 2013-09-04

**Authors:** Tomotada Iwamoto, Louis Grandjean, Kentaro Arikawa, Noriko Nakanishi, Luz Caviedes, Jorge Coronel, Patricia Sheen, Takayuki Wada, Carmen A. Taype, Marie-Anne Shaw, David A. J. Moore, Robert H. Gilman

Some of the data in Table 2 was not displayed correctly. The full, correct table can be viewed here: 

**Figure:**