# Supplementary material for: Genetic Diversity and Transmission Characteristics of Beijing Family Strains of Mycobacterium tuberculosis in Peru
Source: PLoS One. 2012 Nov 21;7(11):e49651. doi: 10.1371/journal.pone.0049651 (PMC3504116; doi:10.1371/journal.pone.0049651)

Figure S1. Quality control approach for VNTR

Basis for the application of capillary electrophoresis system

In the denaturing capillary electrophoresis system, molecular weight of PCR products are indicated as relative migration figure with the size standard. Therefore, there are discrepancy among actual sizes of PCR fragments and apparent sizes indicated by the system differes depending on the locus. However, the differences are highly reproducible among the independent analysis and enable us to create the size offset values for assigning the number of repeats. The reproducibility and accuracy of sizing and the size offsets are checked at every batch of the analysis by including two refence strains (H37Rv and one Beijing strain).

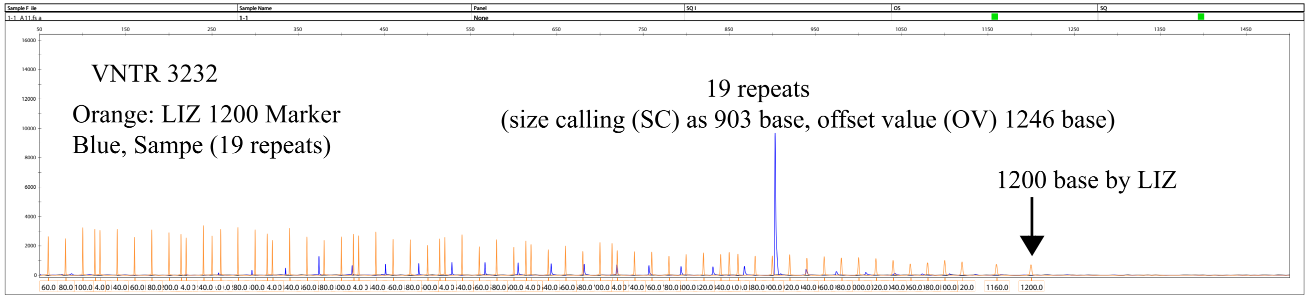

How to assign the large alleles

First, we identify the position of 4 repeats and 15 repeats by the data obtained from H37Rv and control strain which are always included into a batch of the analysis (Most cases we use 96 well plate for a batch of analysis). We, then count the number of stutters to assign the repeat number of the sample at the locus. Here, we can use both offset size value and stutters.

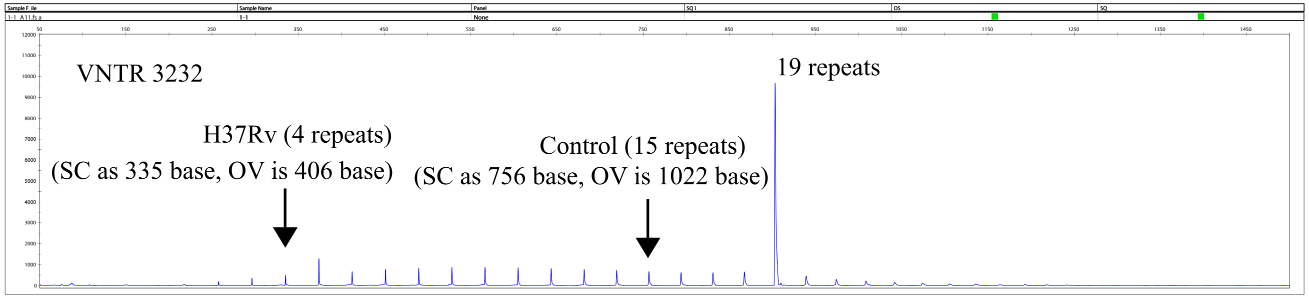

Example of the analysis for 28 repeats

The example of the analysis which was assighned as 28 repeats at VNTR 3232. This is the largest repeat at VNTR 3232 in this study.

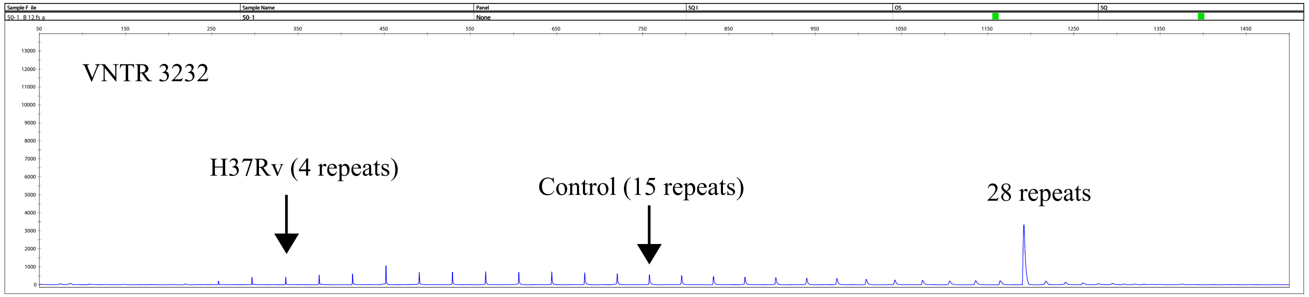

Reproducibility

The results from three independent analysis of the sample with 28 repeat at VNTR 3232 were overlaid on single chart. The data is the evidence for the high reproducibility of size calling and good rational for using off set value. The size variation in an allele is within 5 base.

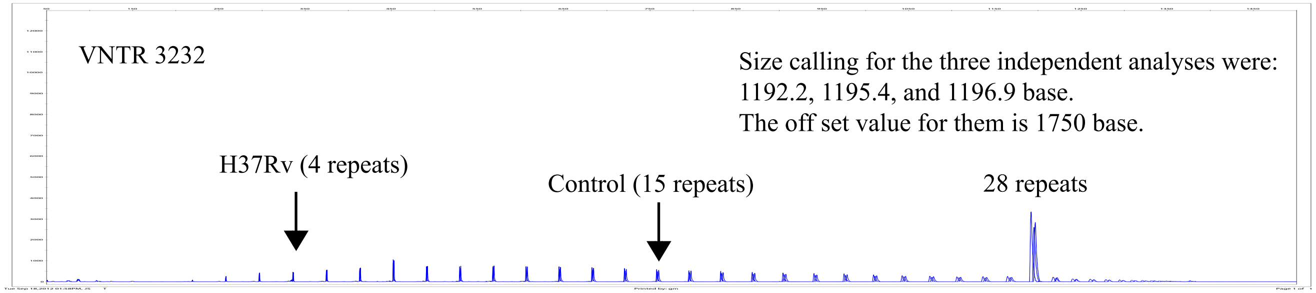

Supplement: Figure S1 — Quality control approach for VNTR. (PDF) [file pone.0049651.s001.pdf]
